# Immediate/Early vs. Delayed Invasive Strategy for Patients with Non-ST-Segment Elevation Acute Coronary Syndromes: A Systematic Review and Meta-Analysis

**DOI:** 10.3389/fphys.2017.00952

**Published:** 2017-11-27

**Authors:** Yanda Li, Zhenpeng Zhang, Xingjiang Xiong, William C. Cho, Dan Hu, Yonghong Gao, Hongcai Shang, Yanwei Xing

**Affiliations:** ^1^Department of Cardiology, Guang'anmen Hospital, Chinese Academy of Chinese Medical Sciences, Beijing, China; ^2^Department of Clinical Oncology, Queen Elizabeth Hospital, Kowloon, Hong Kong; ^3^Masonic Medical Research Laboratory, Utica, NY, United States; ^4^The Key Laboratory of Chinese Internal Medicine of the Ministry of Education, Dongzhimen Hospital Affiliated to Beijing University of Chinese Medicine, Beijing, China

**Keywords:** non-ST-segment elevation acute coronary syndrome (NSTE-ACS), intervention, meta-analysis, major bleeding, invasive strategy, mortality rate

## Abstract

Invasive coronary revascularization has been shown to improve prognoses in patients with non-ST-segment elevation acute coronary syndromes (NSTE-ACS), but the optimal timing of intervention remains unclear. This meta-analysis is to evaluate the outcomes in immediate (<2 h), early (<24 h), and delayed invasive group and find out which is the optimal timing of intervention in NSTE-ACS patients. Studies were identified through electronic literature search of Medline, PubMed Central, Embase, the Cochrane Library, and CNKI. Data were extracted for populations, interventions, outcomes, and risk of bias. All-cause mortality was the pre-specified primary end point. The longest follow-up available in each study was chosen. The odds ratio (OR) with 95% CI was the effect measure. The fixed or random effect pooled measure was selected based on the heterogeneity test among studies. In the comparison between early and delayed intervention, we found that early intervention led to a statistical significant decrease in mortality rate (*n* = 6,624; OR 0.78, 95% CI: 0.61–0.99) and refractory ischemia (*n* = 6,127; OR 0.50, 95% CI: 0.40–0.62) and a non-significant decrease in myocardial infarction (MI), major bleeding and revascularization. In the analysis comparing immediate and delayed invasive approach, we found that immediate intervention significantly reduced major bleeding (*n* = 1,217; OR 0.46, 95% CI: 0.23–0.93) but led to a non-significant decrease in mortality rate, refractory ischemia and revascularization and a non-significant increase in MI. In conclusion, early invasive strategy may lead to a lower mortality rate and reduce the risk of refractory ischemia, while immediate invasive therapy shows a benefit in reducing the risk of major bleeding.

## Introduction

Mortality and incidence of recurrent myocardial infarction (MI) in patients with non-ST-segment elevation acute coronary syndromes (NSTE-ACS) are closely related to public health. Invasive coronary revascularization has been shown to improve outcomes in patients with NSTE-ACS (Mehta et al., 2005; Honnig et al., 2006; Fox et al., 2010), but the optimal timing of intervention remains unclear. The current European Society of Cardiology (ESC) guidelines recommend urgent (defined as <2 h) coronary intervention in patients with a very high risk, defined as: refractory angina, with associated heart failure, life-threatening ventricular arrhythmias, or hemodynamic instability. What is more, early (ESC guidelines definition: <24 h) coronary intervention is recommended in patients with a Global Registry of Acute Coronary Events (GRACE) risk score above 140 or with high-risk features, which is defined as a relevant rise or fall in troponin or dynamic ST or T-wave changes (Hamm et al., 2011; Anderson et al., 2013).

However, at present, the optimal timing of routine invasive intervention is still controversial because of the conflicting reports of existed randomized controlled trials (RCTs) comparing early vs. delayed invasive approaches (Neumann et al., 2003; van't Hof et al., 2003; Mehta et al., 2009; Montalescot et al., 2009; Riezebos et al., 2009; Sciahbasi et al., 2010; Zhang et al., 2010; Thiele et al., 2012; Badings et al., 2013; Tekin et al., 2013; Liu et al., 2015; Reuter et al., 2015; Milosevic et al., 2016; Oosterwerff et al., 2016). Meta-analysis in recent years has also shown insufficient evidence either in favor of or against an early invasive strategy in the NSTE-ACS population (Navarese et al., 2011, 2013; Milasinovic et al., 2015; Jobs et al., 2017). In previous meta-analyses, mortality, MI, refractory ischemia (RI), major bleeding, and repeated revascularization were set as outcomes, and all the data were from the longest available follow-up (Navarese et al., 2011, 2013; Milasinovic et al., 2015; Jobs et al., 2017). Milasinovic D (Milasinovic et al., 2015), Eliano P (Navarese et al., 2011, 2013), and Jobs A (Jobs et al., 2017) found that compared with delayed invasive strategy, early invasive strategy might lead to a significant reduction in the occurrence of refractory ischemia, but conferred no benefit for other outcomes. One of the main limitations of the published meta-analysis was the small number of included studies. After literature retrieval, we updated four new studies (Liu et al., 2015; Reuter et al., 2015; Milosevic et al., 2016; Oosterwerff et al., 2016), hoping to gain a further conclusion with a larger sample size. In addition, by observing the data, we found that several studies used “<2 h” as the timing for immediate invasive therapy (Montalescot et al., 2009; Riezebos et al., 2009; Thiele et al., 2012; Milosevic et al., 2016; Oosterwerff et al., 2016). Therefore, we conducted an updated meta-analysis to assess outcomes between the immediate invasive group and the delayed invasive group.

## Methods

This systematic review and meta-analysis was done in accordance with established methods (Higgins and Green, 2011) and the PRISMA (Preferred Reporting Items for Systematic Reviews and Meta-Analyses) guidelines (Liberati et al., 2009).

### Literature search

**Figure 1:**
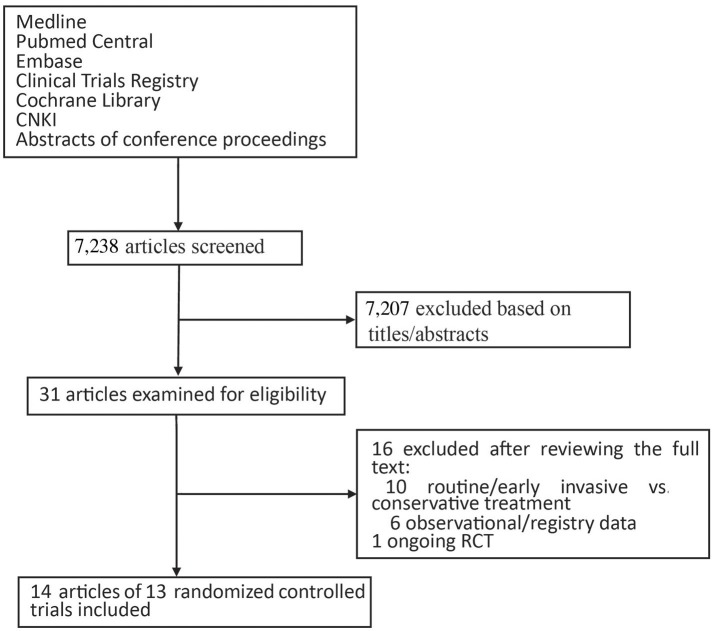
Flow chart of the study selection process.

Studies were identified through a computerized literature search of Medline, PubMed Central, Embase, the Cochrane Library, and CNKI through May 2017. We also searched Clinical Trials Registries (https://clinicaltrials.gov/ and www.controlled-trials.com) for ongoing studies. To providing detailed descriptions of publication screening and reasons for exclusion, the PRISMA flow chart is shown in Figure 1.

### Search strategy

The search strategy terms used were as follows: (Acute coronary syndrome OR unstable angina OR non-stemi OR non-ST elevation) AND (early OR delayed OR late OR immediate OR timing) AND (invasive intervention OR coronary angioplasty OR PCI) AND (randomized controlled trial OR controlled clinical trial OR randomized OR placebo OR clinical trials as topic OR randomly OR trial). Reference lists of all included studies were manually reviewed by independent investigators.

### Selection criteria

Studies were included if they enrolled patients with NSTE-ACS and randomly allocated patients to immediate/early invasive intervention or delayed invasive intervention. Immediate intervention was defined as coronary revascularization less than median 2 h after hospitalization or randomization. Early intervention was defined as coronary revascularization <24 h after enrollment. Delayed intervention was defined as pretreatment using standard medical therapy and subsequent revascularization 24 h or more after enrollment. Mortality rate should be listed as primary outcome. Studies about observational trials or comparing early/routine invasive with conservative/selective invasive strategy or without reliable outcome data were excluded.

### Data extraction

Data were extracted on pre-specified forms by two independent investigators. Internal validity was independently appraised by two investigators, and divergences were resolved by discussion with a third investigator. Clinical characteristics, median time of catheterization and clinical outcomes at follow-up were extracted.

### Quality assessment

**Figure 2:**
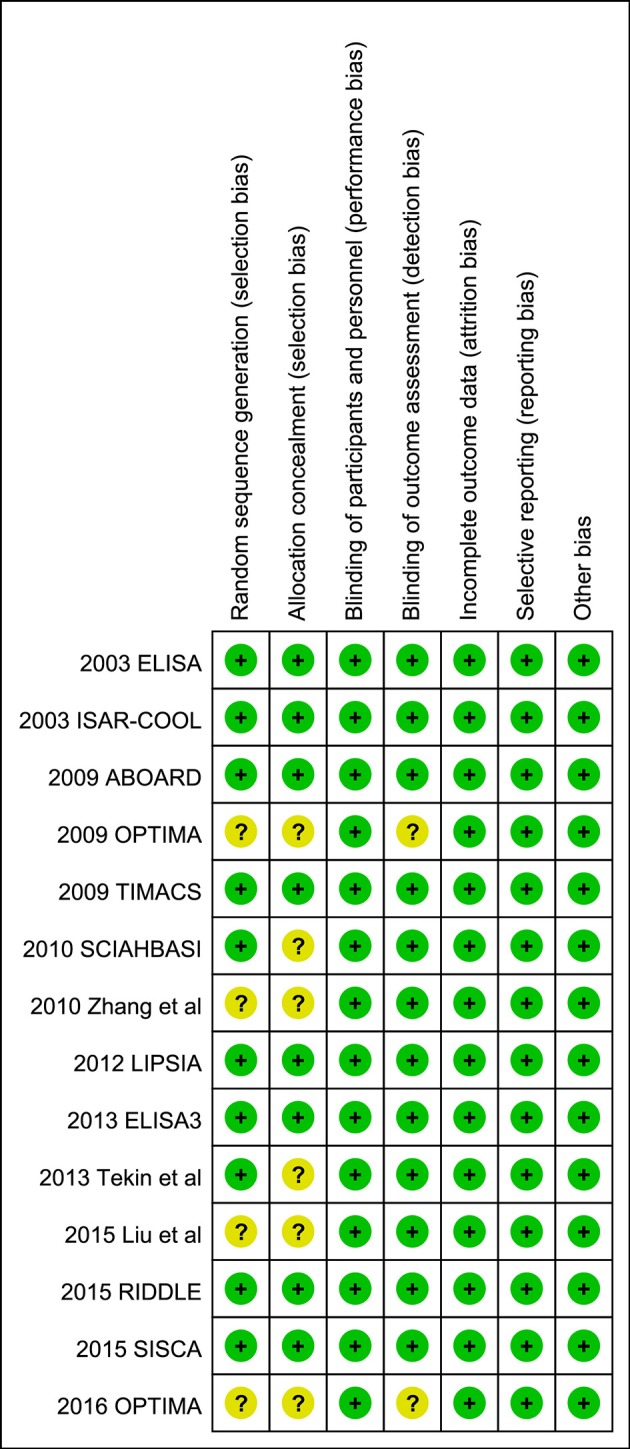
Risk of bias graph: review authors' judgements about each risk of bias item presented as percentages across all included studies.

**Figure 3:**
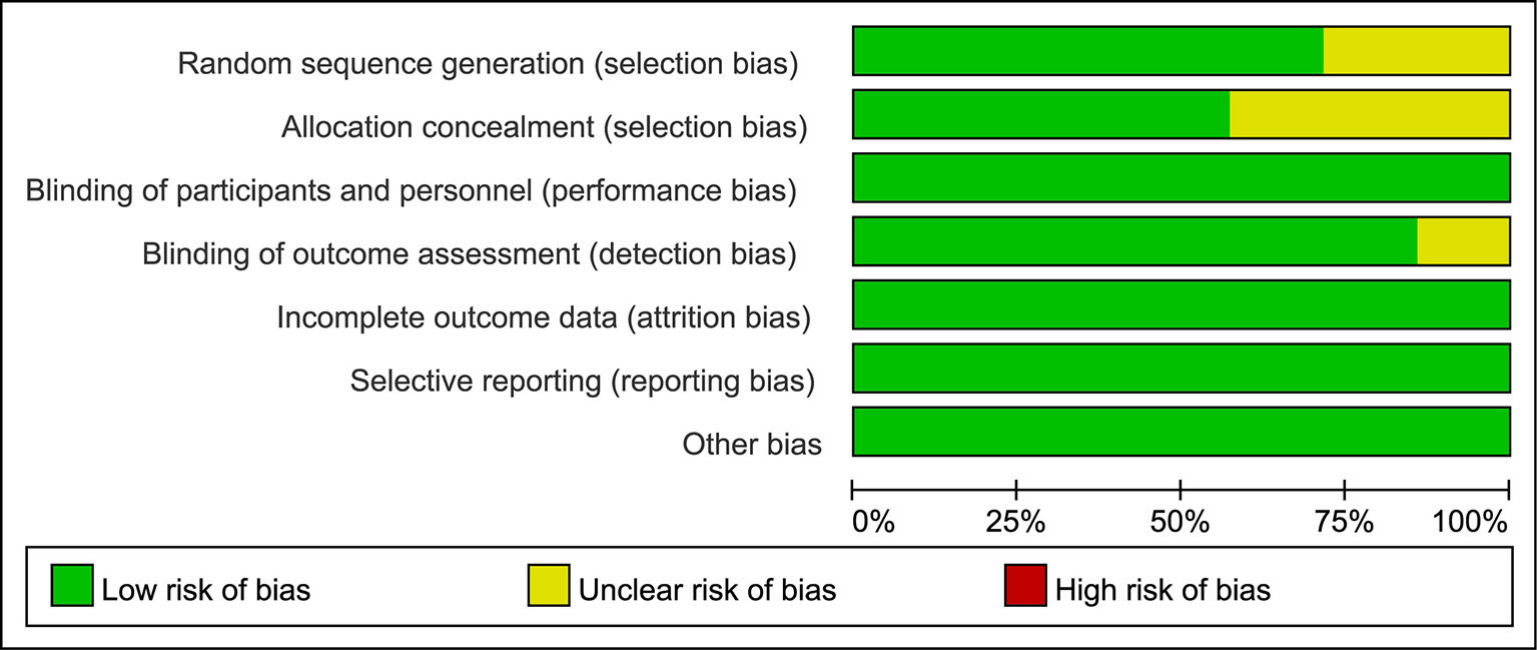
Risk of bias summary: review authors judgements about each risk of bias item for each included study.

The Cochrane Collaboration's tool for assessing risk of bias was used to assess the risk of bias (Higgins and Green, 2011) in the included studies, which is a domain-based evaluation of the following domains: random sequence generation (selection bias); allocation concealment (selection bias); blinding of participants and personnel (performance bias); blinding of outcome assessment (detection bias); incomplete outcome data (attrition bias); selective reporting (reporting bias); and other bias. Assessments for the risk of bias are provided in the risk of bias table for each study (Figures 2, 3).

### Statistical analysis

**Figure 4:**
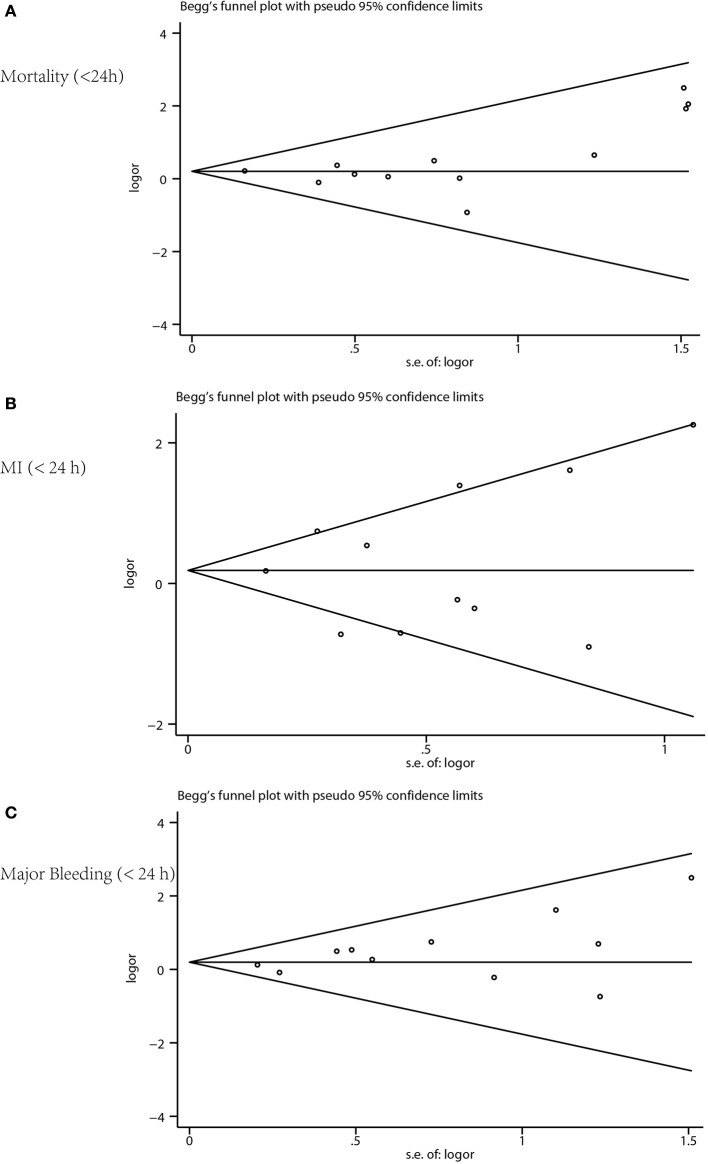
Funnel plot for publication bias. **(A)** Mortality, **(B)** myocardial infarction, and **(C)** major bleeding.

Based on the different timing of the invasive procedures, we conducted two sets of meta-analyses: one in which early invasive therapy (<24 h) was compared with delayed invasive therapy, and another in which immediate invasive therapy (<2 h) was compared with delayed invasive therapy. Data were analyzed according to the intention-to-treat principle. Odds ratios (OR) and 95% confidence interval (CI) were used as summary statistics. The proportion of true variance in estimated effects among the included studies, as opposed to sampling error within studies, was calculated by the *I*^2^ statistic and statistical heterogeneity was considered substantial if *I*^2^ > 75% (Higgins and Green, 2011). Pooled ORs were calculated using the M-H random-effects model and fixed-effects model. Trial Sequential Analysis (TSA) was conducted to evaluate the sample size using TSA software version 0.9.5.9 beta (Supplementary Data Sheet 1). Publication bias was detected using funnel plots, with asymmetry suggesting possible publication bias. Publication bias was also assessed by Begg-test, Egger-test, and the funnel plots made using the STATA software version 12.0 (Figure 4) for the meta-analysis. Publication bias was considered existed if the *P*-value was <0.05. Sensitivity analyses were done by removing one study at a time from the meta-analysis for each of the outcomes using the STATA software version 12.0.

## Results

### Study and patient characteristics

We screened 7,238 potentially relevant articles and excluded 7,201 of them after examining the titles and the abstracts (Figure 1). As a result of reviewing the full texts, we included a total of 14 studies of 13 RCTs; there was one study updated with a 5-year follow-up to the OPTIMA trial first reported in 2009 (Riezebos et al., 2009; Oosterwerff et al., 2016).

The RCTs enrolled 6,624 patients; 3,431 were randomly allocated to early invasive intervention and 3,193 to delayed invasive intervention. Trial characteristics were summarized in Table 1 and additional patient and trial characteristics were shown in Table 2. Baseline characteristics were well-balanced within these studies, including age, female sex, diabetes, and ST-segment depression. The use of glycoprotein IIb/IIIa inhibitors did not differ significantly between different groups. The time of the intervention ranged from 0.5 to 24 h after randomization (early intervention) and 20.5 to 86 h (delayed intervention). The longest available follow-up for all events from the identified publications was recorded and it ranged from 30 days to 5 years. Most patients treated by coronary revascularization had percutaneous coronary intervention (PCI), and some had coronary artery bypass grafting (CABG). Patients randomly assigned to early vs. delayed intervention were well-matched for demographic and clinical characteristics.

**Table 1:** Timing of the invasive approach, definitive treatment, and clinical outcomes at follow-up for included RCTs comparing early and delayed strategies.

**Study, year (References)**	**Median time of catheterization, h**		**Patients**, ***n***		**Definitive treatment (%)**		**Clinical outcomes at follow-up**
	**Early strategy**	**Delayed strategy**	**Early strategy**	**Delayed strategy**	**Early strategy**	**Delayed strategy**	
ELISA, 2003 (van't Hof et al., 2003)	6	50	109	111	PCI:66 (60.5) CABG:15 (13.8) Medical:27 (24.7)	PCI:64 (57.7) CABG:21 (18.9) Medical:26 (23.4)	Death, MI, major bleeding, refractory ischemia at 1 month
ISAR-COOL, 2003 (Neumann et al., 2003)	2.4	86	203	207	PCI:143 (70.4) CABG:16 (7.9) Medical:44 (21.7)	PCI:133 (64.3) CABG:16 (7.7) Medical:58 (28.0)	Death, MI, major bleeding, refractory ischemia at 1 month
OPTIMA, 2009 (Riezebos et al., 2009)	0.5	25	73	69	PCI:73 (100)	PCI:69 (100)	Death, MI, major bleeding, re-PCI at 1 month
OPTIMA, 2016 (Oosterwerff et al., 2016)							Death, MI, re-PCI at 5 years
ABOARD, 2009 (Montalescot et al., 2009)	1.1	20.5	175	177	PCI:117 (66.9) CABG:16 (9.1) Medical:42 (24.0)	PCI:105 (59.3) CABG:17 (9.6) Medical:55 (31.1)	Death, MI, major bleeding, re-PCI, refractory ischemia at 1 month
TIMACS, 2009 (Mehta et al., 2009)	14	50	1593	1438	PCI:954 (59.9) CABG:255 (16.0) Medical:384 (24.1)	PCI:796 (55.4) CABG:219 (15.2) Medical:423 (29.4)	Death, MI, major bleeding, re-PCI, refractory ischemia at 6 months
Zhang et al., 2010 (Zhang et al., 2010)	9.3	49.9	446	369	PCI:314 (70.4) CABG:41 (9.2) Medical:91 (20.4)	PCI:252 (68.3) CABG:37 (10.1) Medical:80 (21.6)	Death, MI, major bleeding, re-PCI, refractory ischemia at 6 months
Sciahbasi, 2010 (Sciahbasi et al., 2010)	5	24	27	27	PCI:27 (100)	PCI:27 (100)	Death, re-PCI in 1 year
LIPSIA-NSTEMI, 2012 (Thiele et al., 2012)	1.1	67.2	200	200	PCI:151 (75.5) CABG:16 (8.0) Medical:33 (16.5)	PCI:114 (57.0) CABG:25 (12.5) Medical:61 (30.5)	Death, MI, refractory ischemia at 6 months, in-hospital major bleeding
ELISA3, 2013 (Badings et al., 2013)	2.6	54.9	269	265	PCI:180 (66.7) CABG:62 (23.2) Medical:27 (10.1)	PCI:164 (61.9) CABG:68 (25.7) Medical:33 (12.4)	Death, MI, major bleeding, refractory ischemia at 1 month
Tekin, 2013 (Tekin et al., 2013)	<24	24–72	69	62	PCI:69 (100)	PCI:62 (100)	Death, MI, LVEF, re-hospitalization at 3 months
RIDDLE, 2015 (Milosevic et al., 2016)	1.4	61	162	161	PCI:127 (78.4) CABG:20 (12.3) Medical:15 (9.3)	PCI:104 (65.0) CABG:38 (23.8) Medical:18 (11.2)	Death, MI, major bleeding, refractory ischemia at 1 year
SISCA, 2015 (Reuter et al., 2015)	2.8	20.9	83	87	PCI:45 (58) CABG:8 (10) Medical:25 (32)	PCI:45 (59) CABG:8 (11) Medical:23 (30)	Death, MI, major bleeding, re-PCI at 1 month
Liu et al., 2015 (Liu et al., 2015)	<12	12–24	22	20	PCI:22 (100)	PCI:20 (100)	Death, MI, major bleeding, re-PCI, refractory ischemia at 6 months

*ABOARD, Angioplasty to Blunt the Rise of Troponin in Acute Coronary Syndromes Randomized for an Immediate or Delayed Intervention; CABG, coronary artery bypass graft; ELISA, Early or Late Intervention in Unstable Angina; ISAR-COOL, Intracoronary Stenting with Antithrombotic Regimen Cooling Off; LIPSIA-NSTEMI, Leipzig Immediate vs. Early and Late Percutaneous Coronary Intervention Trial in Non–ST-Segment Elevation Myocardial Infarction; MI, myocardial infarction; PCI, percutaneous coronary intervention; RIDDLE, Randomized Study of Immediate vs. Delayed Invasive Intervention in Patients with Non-ST-segment Elevation Myocardial Infarction; SISCA, The Invasive Strategy in Acute Coronary Syndrome; TIMACS, Timing of Intervention in Acute Coronary Syndromes*.

**Table 2:** Clinical Characteristics of RCTs in Meta-analysis.

**Study, year (References)**	**Mean age, y**		**Female sex**, ***n*** **(%)**		**Diabetes**, ***n*** **(%)**		**ST-segment depression**, ***n*** **(%)**		**3-vessel disease**, ***n*** **(%)**		**Glycoprotein IIb/IIIa inhibitors**, ***n*** **(%)**	
	**Early strategy**	**Delayed strategy**	**Early strategy**	**Early strategy**	**Early strategy**	**Delayed strategy**	**Early strategy**	**Delayed strategy**	**Early strategy**	**Delayed strategy**	**Early strategy**	**Delayed strategy**
ELISA, 2003 (van't Hof et al., 2003)	63.0	65.0	79 (72.4)	76 (68.4)	16 (14.6)	16 (14.4)	NA	NA	31 (28.4)	33 (29.7)	0 (0.0)	111 (100)
ISAR-COOL, 2003 (Neumann et al., 2003)	70.0	70.0	67 (33.0)	69 (33.3)	53 (26.1)	65 (31.4)	133 (65.5)	135 (65.2)	94 (46.3)	92 (44.4)	203 (100)	207 (100)
OPTIMA, 2009 (Riezebos et al., 2009)/2016 (Oosterwerff et al., 2016)	63.0	62.0	22 (30.0)	19 (26.0)	14 (19.2)	14 (20.3)	38 (52.1)	36 (52.2)	10 (13.7)	9 (13.0)	71 (97.3)	64 (92.8)
ABPARD, 2009 (Montalescot et al., 2009)	65.0	65.0	48 (27.4)	52 (29.4)	38 (21.7)	57 (32.2)	122 (69.7)	136 (76.8)	32 (18.3)	44 (24.9)	114 (65.1)	101 (57.1)
TIMACS, 2009 (Mehta et al., 2009)	65.0	65.7	554 (34.8)	498 (34.6)	422 (26.5)	394 (27.7)	1282 (80.5)	1149 (79.9)	272 (171)	227 (15.8)	370 (23.2)	322 (22.4)
Zhang et al., 2010 (Zhang et al., 2010)	67.0	66.0	151 (33.9)	119 (32.2)	105 (23.5)	83 (22.5)	425 (95.3)	349 (94.6)	195 (43.7)	148 (40.1)	82 (18.4)	79 (21.4)
Sciahbasi, 2010 (Sciahbasi et al., 2010)	58.8	59.7	5 (18.5)	3 (11.1)	7 (26.0)	5 (18.5)	NA	NA	1 (4.0)	2 (7.0)	27 (100)	27 (100)
LIPSIA, 2012 (Thiele et al., 2012)	68.0	70.0	68 (34.0)	72 (36.0)	76 (38.0)	64 (32.0)	122 (61.0)	124 (62.0)	59 (39.5)	63 (31.5)	195 (97.5)	197 (98.5)
ELISA3, 2013 (Badings et al., 2013)	72.1	71.8	82 (30.5)	91 (34.3)	64 (23.8)	54 (20.4)	NA	NA	89 (33.0)	70 (26.6)	NA	NA
Tekin, 2013 (Tekin et al., 2013)	58.1	55.6	28 (40.6)	18 (28.8)	22 (31.9)	28 (45.2)	56 (81.2)	50 (80.6)	40 (58.1)	35 (56.5)	NA	NA
RIDDLE, 2015 (Milosevic et al., 2016)	60.5	63.0	48 (29.6)	55 (34.2)	35 (21.6)	52 (32.3)	125 (77.7)	130 (80.7)	57 (35.2)	65 (40.6)	152 (93.8)	146 (90.7)
SISCA, 2015 (Reuter et al., 2015)	63.9	66.5	25 (30.0)	23 (27.0)	35 (42.0)	28 (33.0)	63 (76.0)	65 (76.0)	NA	NA	83 (100)	87 (100)
Liu et al., 2015 (Liu et al., 2015)	80.4	80.3	11 (50.0)	7 (35.0)	11 (50.0)	11 (55.0)	NA	NA	7 (31.8)	9 (45.0)	NA	NA

### Risk of bias

A review of the authors' judgments about each risk of bias item showed as percentages across all of the included RCTs. The quality of the selected studies was assessed according to the Cochrane criteria (Figures 2, 3).

The RCTs were similar in their risk of bias. All were done according to the intention-to-treat principle; losses to follow-up were rare and described in detail. However, allocation concealment was not clearly addressed in six studies (Figure 2). Funnel plot for mortality, MI, and major bleeding revealed no apparent asymmetry, thus minimizing a potential risk of publication bias (Figure 4). Egger's regression test of publication bias was shown in Table 3 and indicated little evidence of publication bias.

**Table 3:** Egger's test of publication bias for mortality, MI, and major bleeding.

**Outcomes**	**Std. Eff**.	**Coef**.	**Std. Err**.	***t***	***P* > *t***	**[95% CI]**	
Mortality (<24 h)	Slope	0.0462328	0.1642426	0.28	0.784	−0.3197226	−0.4121883
	Bias	0.4695734	0.3770398	1.25	0.241	−0.3705238	1.30967
MI (<24 h)	Slope	0.0716176	0.3936487	0.18	0.860	−0.8188776	0.9621127
	Bias	0.3686991	1.099424	0.34	0.745	−2.118372	2.85577
Major bleeding (<24 h)	Slope	−0.0947393	0.181002	−0.52	0.613	−0.5041942	0.3147156
	Bias	0.7986756	0.4080998	1.96	0.082	−0.1245103	1.721861

*Coef, coefficient; Std. Eff, Standard Effect; Std. Err, Standard Error*.

### Early (<24 h) invasive therapy vs. delayed invasive therapy

**Figure 5:**
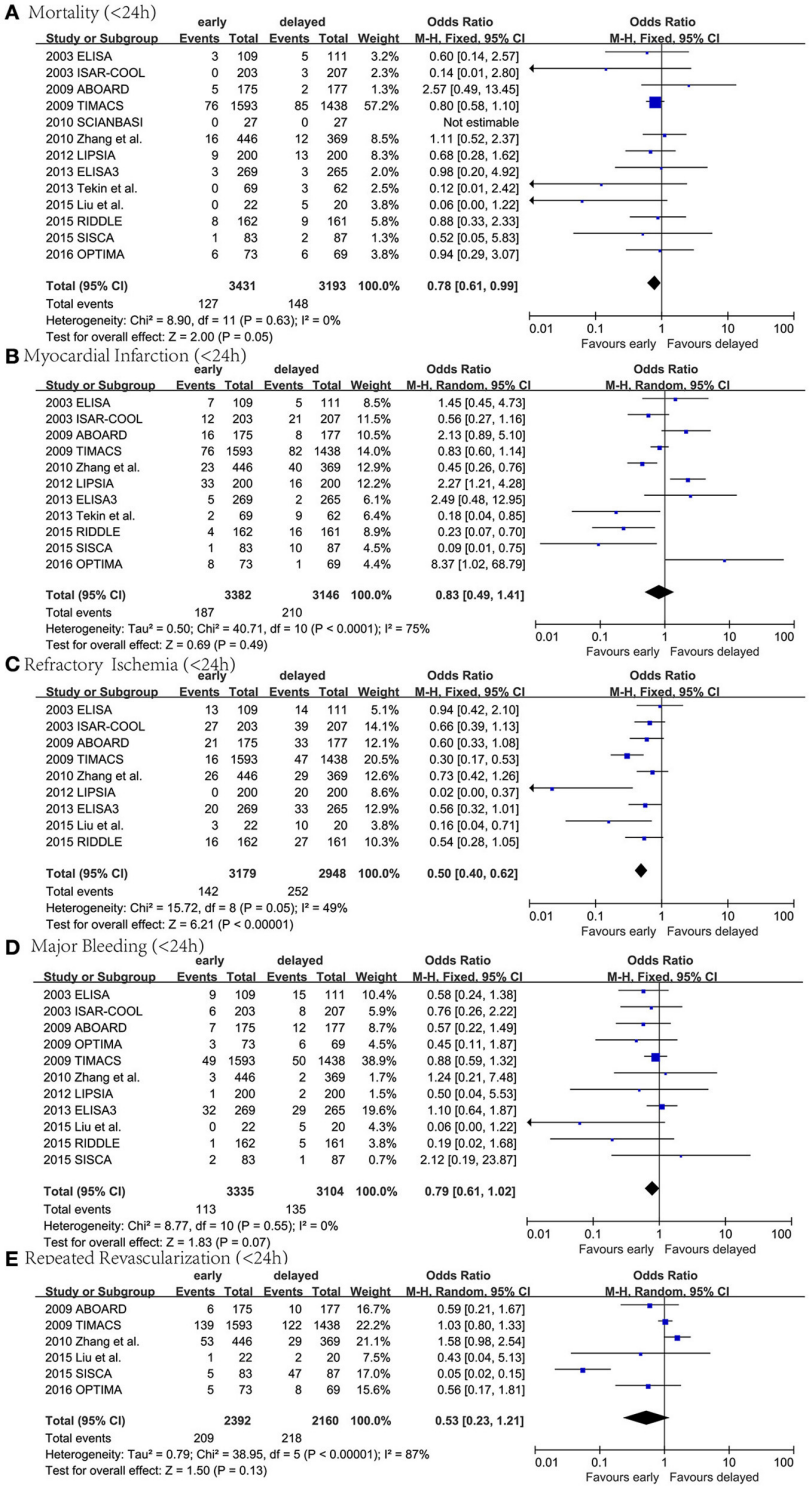
Forest plots comparing outcomes between early (<24 h) invasive group and delayed invasive group. **(A)** Mortality, **(B)** myocardial infarction, **(C)** refractory Ischemia, **(D)** major bleeding, and **(E)** repeated revascularization.

Individual and pooled ORs for mortality, MI, RI, major bleeding, and repeated revascularization are shown in Figure 5. Thirteen RCTs reported mortality, the results of the meta-analysis showed a statistically significant difference (*n* = 6,624; OR 0.78, 95% CI: 0.61–0.99; *I*^2^ = 0%; *P* = 0.05; Figure 5A). Eleven RCTs reported MI, the results of the meta-analysis showed no statistically significant difference (*n* = 6,528; OR 0.83, 95% CI: 0.49–1.41; *I*^2^ = 75%; *P* = 0.49; Figure 5B). Nine RCTs reported RI. The results of the meta-analysis showed that early invasive therapy was associated with a significant decrease in RI compared to the delayed invasive group (*n* = 6,127; OR 0.50, 95% CI: 0.40–0.62; *I*^2^ = 49%; *P* < 0.00002; Figure 5C). Eleven RCTs reported major bleeding. The results of the meta-analysis showed no statistically significant difference (*n* = 6,439; OR 0.79, 95% CI: 0.61–1.02; *I*^2^ = 0%; *P* = 0.07; Figure 5D). Six RCTs reported repeated revascularization. The results of the meta-analysis showed no statistically significant difference (*n* = 4,553; OR 0.53, 95% CI: 0.23–1.21; *I*^2^ = 87%; *P* = 0.13; Figure 5E).

### Immediate (<2 h) invasive therapy vs. delayed invasive therapy

**Figure 6:**
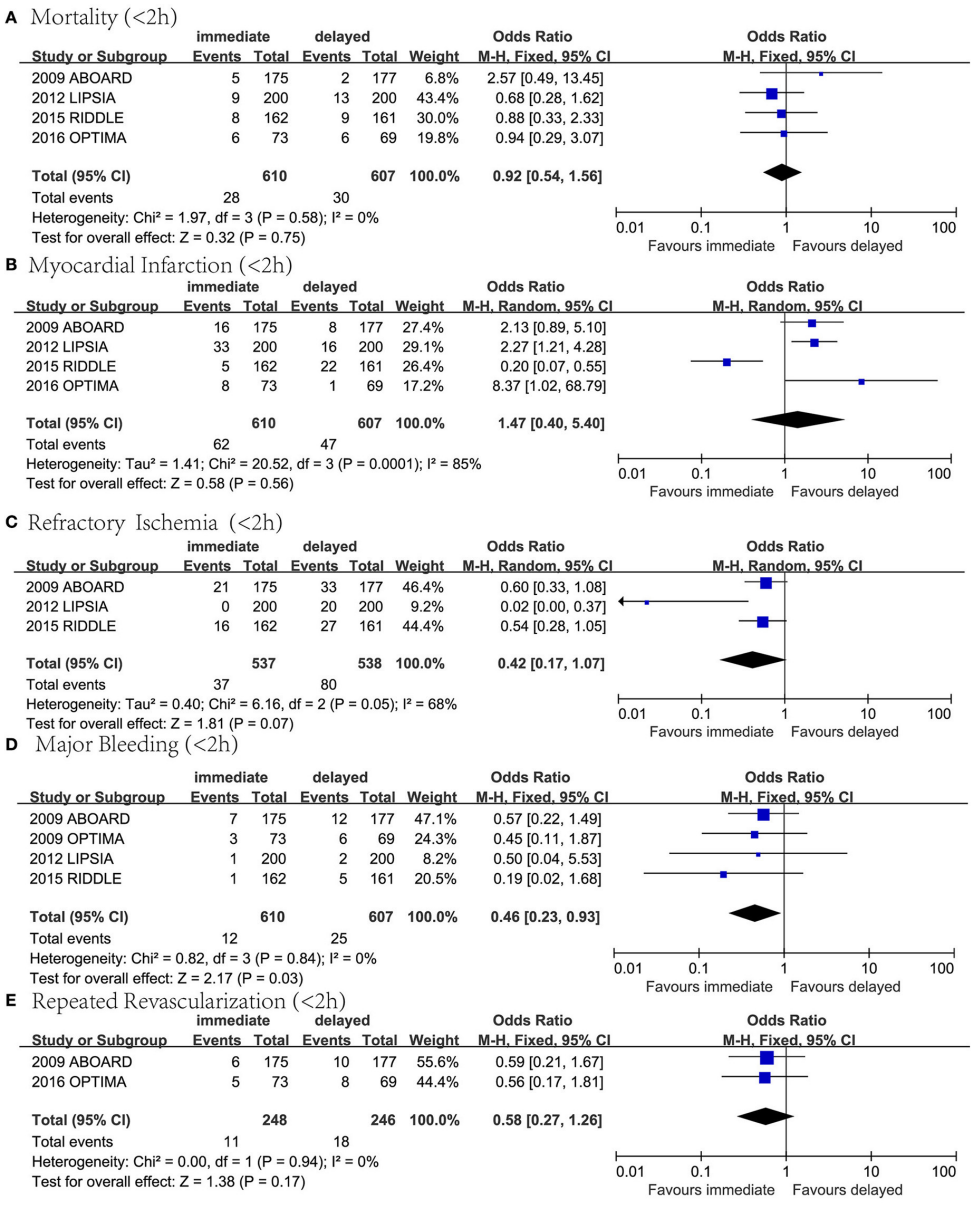
Forest plots comparing outcomes between immediate (<2) invasive group and delayed invasive group. **(A)** Mortality, **(B)** myocardial infarction, **(C)** refractory ischemia, **(D)** major bleeding, and **(E)** repeated revascularization.

Individual and pooled ORs for mortality, MI, RI, major bleeding and repeated revascularization are shown in Figure 6. Four RCTs reported mortality. The results of the meta-analysis showed no statistically significant difference (*n* = 1,217; OR 0.92, 95% CI: 0.54–1.56; *I*^2^ = 0%; *P* = 0.75; Figure 6A). Four RCTs reported MI. The results of the meta-analysis showed no statistically significant difference (*n* = 1,217; OR 1.47, 95% CI: 0.40–5.40; *I*^2^ = 85%; *P* = 0.56; Figure 6B). We excluded individual studies to conduct sensitivity analyses to illustrate the heterogeneity, and the results showed a significant difference when the data from RIDDLE was excluded (95% CI 1.45–3.94; *P* = 0.0006; *I*^2^ = 0%). Three RCTs reported RI. The results of the meta-analysis showed no statistically significant difference (*n* = 1,075; OR 0.42, 95% CI: 0.17–1.07; *I*^2^ = 68%; *P* = 0.07; Figure 6C). Four RCTs reported major bleeding. The results of the meta-analysis showed that immediate invasive therapy could lead to a significant decrease in major bleeding compared to the delayed invasive group (*n* = 1,217; OR 0.46, 95% CI:0.23–0.93; *I*^2^ = 0%; *P* = 0.03; Figure 6D). Two RCTs reported repeated revascularization. The results of the meta-analysis showed no statistically significant difference (*n* = 494; OR 0.58, 95% CI: 0.27–1.26; *I*^2^ = 0%; *P* = 0.17; Figure 6E).

### Sensitivity analyses

**Figure 7:**
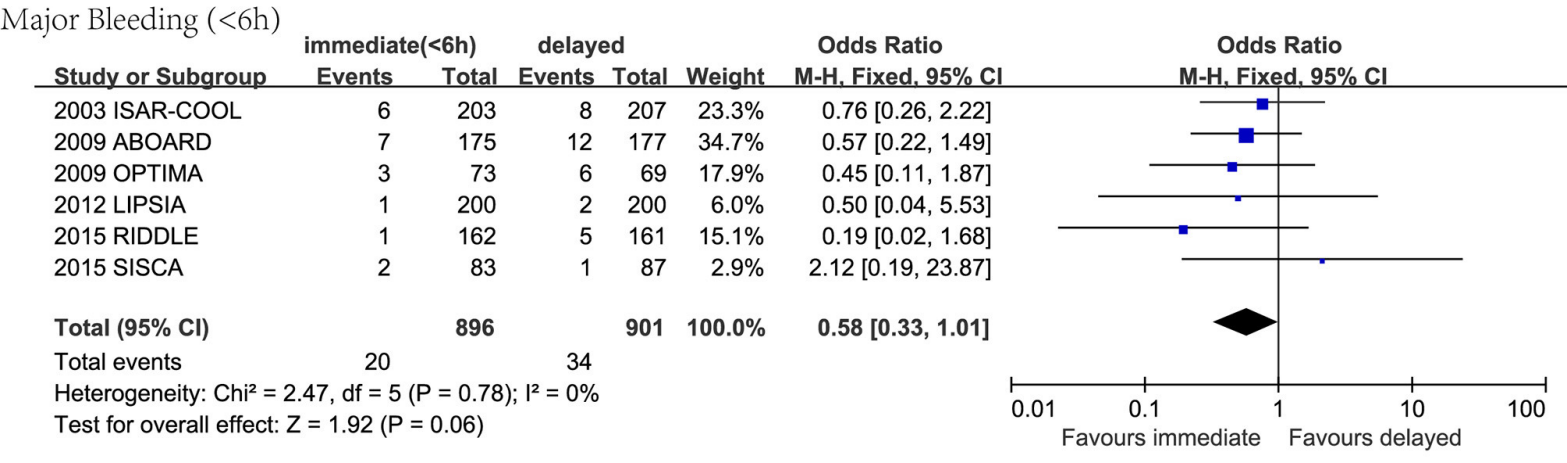
Forest plot comparing major bleeding between immediate (<6 h) invasive group and delayed invasive group.

Sensitivity analyses were done by removing one study at a time from the meta-analysis for each of the outcomes (Supplementary Data Sheet 1). We found that in the comparison assessing mortality rate, MI, major bleeding and repeated revascularization between early and delayed invasive group (Figure 5), in the comparison assessing major bleeding and repeated revascularization between the immediate (<2 h) and delayed invasive group (Figure 6), and in the comparison assessing major bleeding and repeated revascularization between the immediate (<6 h) and delayed invasive group (Figure 7), exclusion of the data from several RCTs significantly skewed the results, indicating that these reports might be statistically unstable.

### Trial sequential analysis

**Figure 8:**
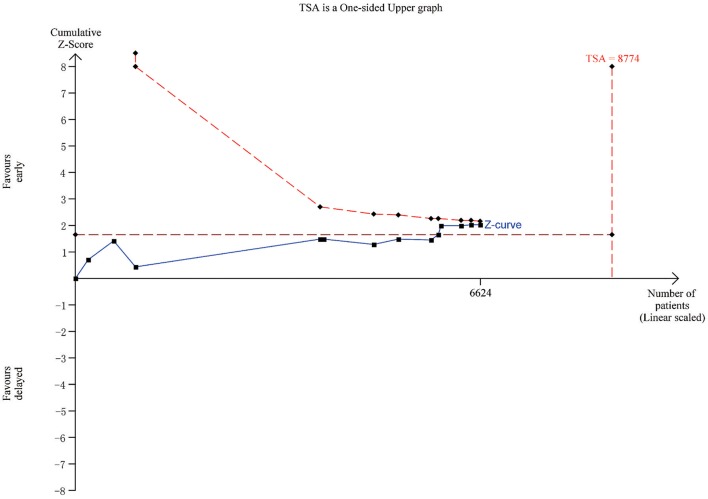
TSA plot for the comparison assessing mortality rate between early (<24 h) and delayed invasive therapy.

TSA was done to evaluate the sample size and correct errors in the comparison assessing mortality rate between early (< 24 h) and delayed invasive therapy (Figure 8). The Lan-DeMets sequential monitoring boundary, which assumes a 6% control event rate and a 20% relative risk reduction with 80% power, has not been crossed, indicating that the cumulative evidence is inconclusive.

## Discussion

We included 14 studies to analyze outcomes among patients with NSTE-ACS receiving immediate/early compared with delayed invasive approach and found that early intervention led to a statistical significant decrease in mortality rate and refractory ischemia and a non-significant decrease in MI, major bleeding and revascularization, while immediate invasive therapy was associated with a reduction in major bleeding.

### Added value to previous meta-analyses on the same topic

We updated four high-quality RCTs for the invasive strategy in NSTE-ACS. Although this was an updated meta-analysis, new results and content were found to provide evidence for the update of the guidelines. Compared with published meta-analyses, we divided the definition of early intervention into immediate (<2 h) and early (<24 h), since timing of intervention would play an important role on clinical endpoints. We divided the meta-analysis in two components, one comparing early (<24 h) vs. delayed (> 24 h) and the other one comparing immediate (<2 h) vs. delayed, so as to seek more accurate conclusions on the impact of revascularization timing if NTSE-ACS on adverse events. Through comparison and analysis, we found early intervention led to a statistical significant decrease in mortality rate and immediate vs. delayed reduced the risk of major bleeding. Compared with other published (Rajpurohit et al., 2013), this result was first found in our article. Besides, in RCTs published before 2015, there was only the TIMACS trial, which used GRACE score as risk stratification. And the current guidelines recommend urgent coronary angiography in patients with a very high risk (GRACE score > 140), this is totally based on the conclusion of the TIMACS trial. However, in our updated analysis, we found the RIDDLE-NSTEMI trial also made a subgroup analysis, which showed no significant difference on the primary endpoint between patients in immediate and delayed invasive group, no matter the GRACE risk score was above 140 or below 140. We found another large sample study compared immediate and delayed intervention in NSTE-ACS patients: the IDEAL NSTEMI study (cinicaltrials.gov identifier NCT01638806; IDEAL NSTEMI, 2012), which will be completed in recent years, we will keep focus on this trial and update our conclusions at the first time.

### Early invasive strategy might reduce mortality rate

In the comparison assessing mortality rate between early and delayed invasive strategy, we found that early intervention led to a statistical significant decrease in mortality rate (*n* = 6,624; OR 0.78, 95% CI: 0.61–0.99; *I*^2^ = 0%; *P* = 0.05; Figure 5A). This result was firstly found, the *P*-value of the assessment of mortality was 0.17 in 2011, 0.18 in 2013, 0.16 in 2015 and 0.0879 in 2016 (Navarese et al., 2011, 2013; Milasinovic et al., 2015; Jobs et al., 2017), and with the inclusion of four new RCTS, a significant difference of the primary outcome appeared. Besides, the heterogeneity was low (*I*^2^ = 0%), which increased the credibility of this result. However, the upper limit value of CI was very close to 1 and *P* was the critical value, and according to the result of TSA, as the boundary line was not crossed, this conclusion was still inconclusive (Figure 8), to gain a stable conclusion, further RCTs of large sample size and long-term follow-up are warranted.

The debate about the optimal timing of intervention for NSTE-ACS patients have lasted for years. On the one hand, an early approach may facilitate rapid diagnosis, earlier revascularization, and shorter hospital stays; on the other hand, patients may also take potential risks because of intervention on unstable plaques with fresh thrombus. Conversely, an optimal medical treatment for plaque passivation in delayed strategy may lead to benefits through following intervention on more stable plaques. Based on the results, it seems that early invasive strategy could provide a higher survival rate, and the potential benefit of delayed strategy might be offset. This may due to a higher risk for events while waiting for angiography.

### Immediate invasive strategy might reduce the risk of major bleeding

In the analysis comparing immediate (within a median of 2 h after randomization) and delayed invasive approach, it is worth mentioning that immediate intervention significantly reduced major bleeding and the heterogeneity was low (*n* = 1,217; OR 0.46, 95% CI:0.23–0.93; *I*^2^ = 0%; *P* = 0.03; Figure 6D). As to further specify the influence of the timing on major bleeding, we conducted a new analysis including trials in which intervention was done within 6 h after randomization and the result showed a non-significant decrease in the risk of major bleeding (*n* = 1,797; OR 0.58, 95% CI: 0.33–1.01; *I*^2^ = 0%; *P* = 0.06; Figure 7).

In addition, we found that immediate approach led to a non-significant decrease in mortality rate, RI and revascularization and a non-significant increase in MI. Combing with the data in the comparison between early and delayed intervention group, this result suggested that the risk of major bleeding might increase with the delay of the timing of intervention: intervention within 2 h significantly reduced the risk of major bleeding; in the case of intervention within 6 h, there was a weak tendency toward less bleeding (*P*-value = 0.06); when assessing the effect of intervention within 24 h, this advantage had almost disappeared (the *P*-value was 0.55).

In the four trails defining “<2 h” as immediate, antithrombotic pre-treatment was approximate: dual antiplatelet therapy with aspirin and clopidogrel was used, along with heparin. Abciximab (0.25 mg/kg followed by an infusion of 10 μg/min for 12 h) was used in OPTIMA and ABOARD, while tirofiban (25 μg/kg followed by continuous infusion of 0.15 μg/kg/min for 24 h) was used in LIPSIA. However, part of the description of the dose of medication was not very clear, some drugs were used “for at least 24 h” and some were “left to the discretion of the investigators.” The risk of major bleeding decreased in immediate invasive group, one possible reason was that if the intervention was immediately started, the dosage of drugs that may cause bleeding complications (such as heparin and Glycoprotein IIb/IIIa inhibitors) was considerably reduced, as these antiplatelet drugs would be used throughout the waiting period of surgery.

The common sources of non-access site-related bleeding after PCI included gastrointestinal bleeding, cerebral hemorrhage, retroperitoneal hematoma, etc. (Kwok et al., 2015). Gastrointestinal bleeding had been associated with an in-hospital mortality of ≤10% (Abbas et al., 2005; Nikolsky et al., 2009). The onset of MI might cause fear, anxiety and pain, stimulate the sympathetic nerves and increase catecholamine secretion, constrict gastric mucosal vessel and lead to acute gastric mucosal bleeding. So, another possible reason was that the waiting time of patients in immediate intervention group was shortened, the remission of MI symptoms relieved the patients' anxiety and physical pain, thus lessening the risk of stress ulcer, which perhaps was the main cause of gastrointestinal bleeding after intervention (Tetsuya et al., 2011).

### Other outcomes

In the comparison between early (within 24 h after randomization) and delayed intervention, we found that early intervention led to no significant decrease in MI, major bleeding, and revascularization and a statistically significant decrease in refractory ischemia, which was supported by previous pooled meta-analysis.

### Risk stratification

The current guidelines recommend urgent (ESC guidelines definition: <2 h) coronary intervention in patients with a very high risk, defined as: refractory angina, with associated heart failure, life-threatening ventricular arrhythmias, or hemodynamic instability. In addition, early (ESC guidelines definition: <24 h) coronary angiography is recommended to patients whose GRACE risk score is above 140 or with high-risk features, defined as a relevant rise or fall in troponin or dynamic ST or T-wave changes (Hamm et al., 2011; Levine et al., 2011). However, only 2 of 14 trails used GRACE score as risk stratification. One was the TIMACS trial, the other was the RIDDLE-NSTEMI trial which was updated by our meta-analysis. In the TIMACS trial, In patients with a GRACE risk score of more than 140 (the highest risk), the mortality rate was 13.9% in the early-intervention group, as compared with 21.0% in the delayed-intervention group, a reduction of 35.0% in the early-intervention group (HR, 0.65; 95% CI, 0.48–0.89; *P* = 0.006). However, among patients with a score of 140 or less (a combination of the low risk), no significant difference between the two groups was found (7.6 vs. 6.7%; hazard ratio, 1.12; 95% CI, 0.81–1.56; *P* = 0.48; *P* = 0.01 for heterogeneity; Mehta et al., 2009). However, in the RIDDLE-NSTEMI trial, there was no significant difference on the primary endpoint between patients in immediate and delayed invasive group, no matter the GRACE risk score was above 140 or below 140 (Milosevic et al., 2016). Although these two articles used the same hierarchical approach with GRACE score, the RIDDLE-NSTEMI trial and the TIMACS trial reached different conclusions. In the RIDDLE-NSTEMI trial, there was no significant interaction between the two groups denoting pre-specified subgroups and the assignment to immediate vs. delayed invasive strategy on the primary endpoint (Milosevic et al., 2016). However, in the TIMACS trial, early invasive strategy did not differ greatly from delayed intervention in preventing the primary outcome, but in high-risk patients, it did reduce the rate of the composite secondary outcome of death, MI, or refractory ischemia and was superior to delayed intervention (Mehta et al., 2009). Current recommendations from international guidelines for NSTE-ACS are based on the subgroup analysis of the TIMACS trial. However, the only two articles for classification with a GRACE risk score led to different conclusions. Further large-scale, high-quality RCTs with adequate follow-up duration as well as risk stratification analysis are warranted.

### Definition of new MI

In NSTE-ACS patients, refractory ischemia has been associated with a more than 4-fold risk of developing MI (Mehta et al., 2009), however, the reduction of refractory ischemia in early intervention group was not translated into lower MI rates and the heterogeneity was high (*I*^2^ = 75% and *I*^2^ = 85% in Figures 5B, 6B, respectively), it was probably because the definition of new MI was not consistent among these studies. In RCTs of past few years, the definition was relatively more reliable. For example, in RIDDLE, the definition of new MI was associated with the time period after randomization (Mehta et al., 2009). But in some studies such as OPTIMA, the definition solely based on the rise in CK-MB, this non-standard definition may have led to overestimation of the new MI rate. If different definitions were used on the same set of patients, the rates of MI reported would be significantly different, therefore, the benefit of early intervention in NSTE-ACS patients might be underestimated.

### Limitations

On a whole, the overall sample size is small, especially in the comparison between immediate and delayed intervention groups; although immediate intervention showed a benefit in decreasing of major bleeding, there were only four included studies and thus the results should be treated with caution. Further data from high-quality RCTs are required to reach reliable conclusions. Besides, the event rates was low and a single trial (TIMACS) contributed to most events. Furthermore, we found that the heterogeneity in the timing of intervention and in patient risk profiles. Besides, most of the follow-up periods of these RCTs were <6 months, only one study (OPTIMA) conducted a follow-up to 5 years, more RCTs with long term follow-up were still warranted. Nevertheless, this study is the largest pooled data sample of RCTs on early/immediate vs. delayed invasive intervention in NSTE-ACS populations and offers basic knowledge for future clinical trials.

## Conclusions

For the first time, it was found that compared with a delayed invasive strategy, an early invasive strategy might reduce mortality rate and the risk of refractory ischemia. However, as the upper limit value of CI was very close to 1 and *P* was the critical value, and the result of TSA indicated that the meta-analysis may result in false positive. Meanwhile, immediate invasive therapy could reduce the risk of major bleeding. To achieve definitive conclusion, RCTs of large sample size, long-term follow-up and clinically relevant definition of periprocedural MI are warranted.

## Author contributions

YX and XX: defined the research theme; YL, WC, ZZ, YG, and DH: designed the methods and analyzed the data; HS, YL, and YX: interpreted the results; YX and YL: wrote the manuscript; All authors discussed the results and commented on the manuscript.

### Conflict of interest statement

The authors declare that the research was conducted in the absence of any commercial or financial relationships that could be construed as a potential conflict of interest.
