# Supplementary material for: Immediate/Early vs. Delayed Invasive Strategy for Patients with Non-ST-Segment Elevation Acute Coronary Syndromes: A Systematic Review and Meta-Analysis
Source: Front Physiol. 2017 Nov 27;8:952. doi: 10.3389/fphys.2017.00952 (PMC5712112; doi:10.3389/fphys.2017.00952)
Supplement: Supplementary file 1 [file DataSheet1.docx]

**Figure.1 Sensitivity analysis plot of mortality rate between early invasive strategy and delayed invasive strategy.**


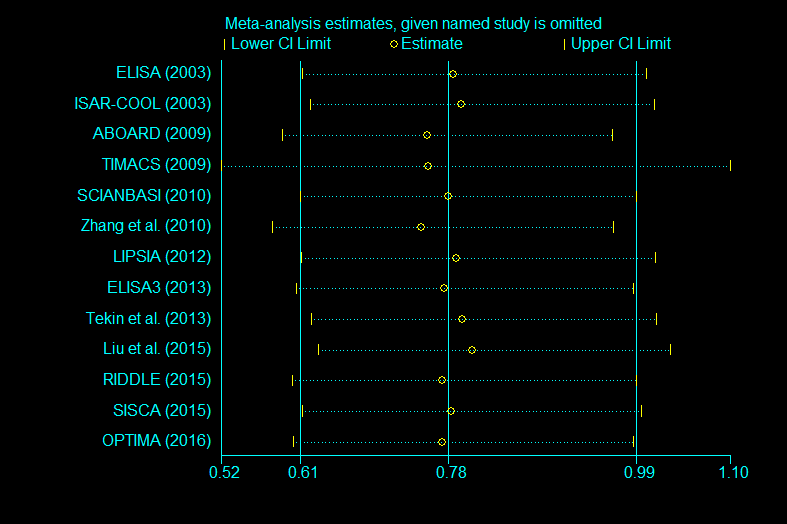


|  |  |  |  |  |  |  |
| --- | --- | --- | --- | --- | --- | --- |
| ------------------------------------------------------------------ | | | | | | |
| Study omitted \| Estimate [95% Conf. Interval] | | | | | | |
| -------------------+---------------------------------------------- | | | | | | |
| ELISA (2003) \| .78715879 .61590993 1.006022 | | | | | | |
| ISAR-COOL (2003) \| .79626393 .62421203 1.0157386 | | | | | | |
| ABOARD (2009) \| .75752479 .59267294 .96823019 | | | | | | |
| TIMACS (2009) \| .75926113 .52309221 1.1020571 | | | | | | |
| SCIANBASI (2010) \| .78109908 .61330312 .99480301 | | | | | | |
| Zhang et al. (2010)\| .75079137 .58143425 .96947795 | | | | | | |
| LIPSIA (2012) \| .79050052 .61455804 1.0168138 | | | | | | |
| ELISA3 (2013) \| .7769208 .60831565 .99225783 | | | | | | |
| Tekin et al. (2013)\| .79770058 .62529463 1.0176421 | | | | | | |
| Liu et al. (2015) \| .80932331 .63390952 1.0332772 | | | | | | |
| RIDDLE (2015) \| .77520895 .60397732 .99498594 | | | | | | |
| SISCA (2015) \| .7845512 .61522216 1.0004851 | | | | | | |
| OPTIMA (2016) \| .77480245 .60516959 .99198443 | | | | | | |
| -------------------+---------------------------------------------- | | | | | | |
| Combined \| .78109909 .61330313 .994803 | | | | | | |
| ------------------------------------------------------------------ | | | | | | |

**Figure.2 Sensitivity analysis plot of myocardial infarction between early invasive strategy and delayed invasive strategy.**


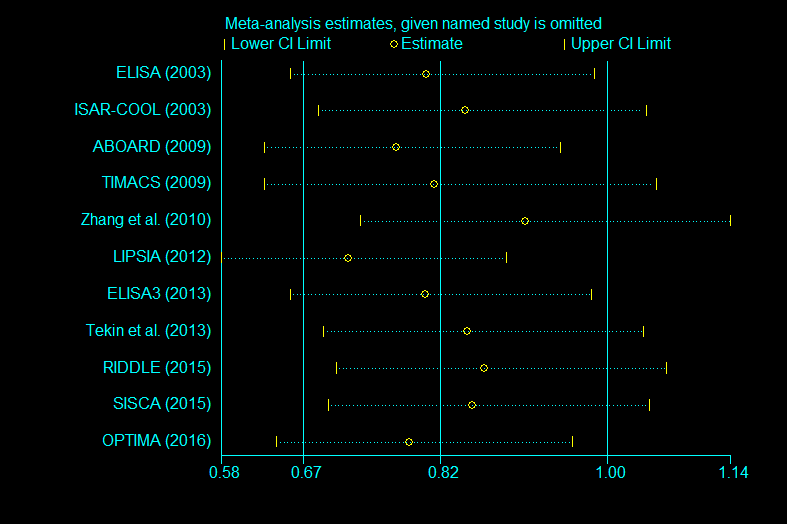


| ------------------------------------------------------------------------------ |
| --- |
| Study omitted \| Estimate [95% Conf. Interval] |
| -------------------+---------------------------------------------------------- |
| ELISA (2003) \| .80477506 .65464133 .98934007 |
| ISAR-COOL (2003) \| .84704918 .68541998 1.0467923 |
| ABOARD (2009) \| .77187347 .62559462 .95235574 |
| TIMACS (2009) \| .8134225 .62549585 1.0578107 |
| Zhang et al. (2010)\| .91350055 .7322166 1.1396672 |
| LIPSIA (2012) \| .71854192 .57809061 .89311683 |
| ELISA3 (2013) \| .80320793 .65417045 .98620015 |
| Tekin et al. (2013)\| .84956348 .69143987 1.043848 |
| RIDDLE (2015) \| .86799347 .70482671 1.0689331 |
| SISCA (2015) \| .85510558 .69604546 1.0505141 |
| OPTIMA (2016) \| .78568554 .63947612 .96532422 |
| -------------------+---------------------------------------------------------- |
| Combined \| .81942204 .66875533 1.0040331 |
| ------------------------------------------------------------------------------ |

**Figure.3 Sensitivity analysis plot of refractory ischemia between early invasive strategy and delayed invasive strategy.**


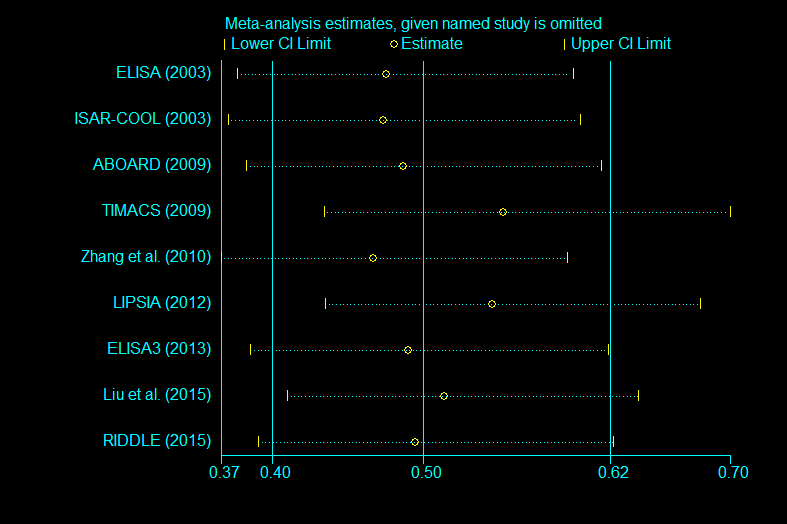


| ------------------------------------------------------------------------------ |
| --- |
| Study omitted \| Estimate [95% Conf. Interval] |
| -------------------+---------------------------------------------------------- |
| ELISA (2003) \| .47919366 .38227892 .60067809 |
| ISAR-COOL (2003) \| .47688022 .37594333 .60491765 |
| ABOARD (2009) \| .49001533 .38805777 .61876106 |
| TIMACS (2009) \| .55510974 .43841201 .70287049 |
| Zhang et al. (2010)\| .47073114 .37133151 .59673846 |
| LIPSIA (2012) \| .54793668 .43930626 .68342888 |
| ELISA3 (2013) \| .4935599 .39067519 .62353933 |
| Liu et al. (2015) \| .51637942 .41455698 .64321119 |
| RIDDLE (2015) \| .49805534 .39584336 .62665981 |
| -------------------+---------------------------------------------------------- |
| Combined \| .50275459 .40469901 .62456833 |
| ------------------------------------------------------------------------------ |

**Figure.4 Sensitivity analysis plot of major bleeding between early invasive strategy and delayed invasive strategy.**


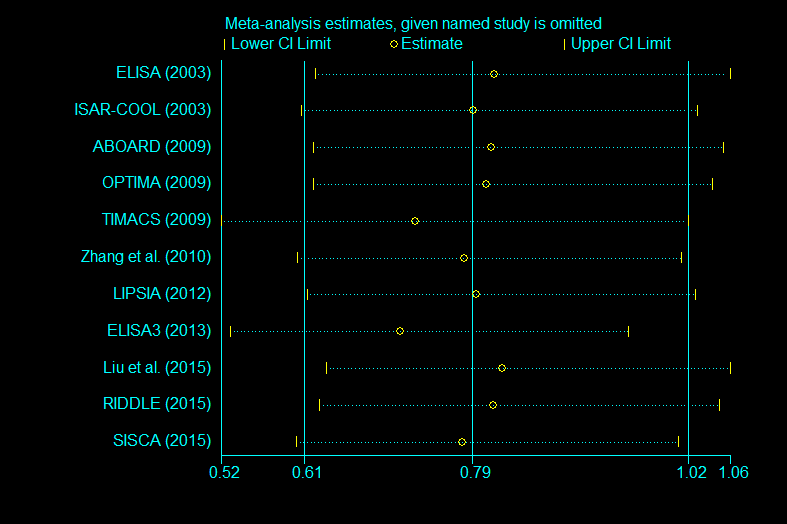


| ------------------------------------------------------------------------------ |
| --- |
| Study omitted \| Estimate [95% Conf. Interval] |
| -------------------+---------------------------------------------------------- |
| ELISA (2003) \| .81082499 .6191324 1.0618685 |
| ISAR-COOL (2003) \| .78819335 .60463703 1.0274739 |
| ABOARD (2009) \| .8068381 .61741203 1.0543814 |
| OPTIMA (2009) \| .80229616 .61735719 1.0426365 |
| TIMACS (2009) \| .72629541 .51862431 1.0171237 |
| Zhang et al. (2010)\| .77870584 .60024655 1.0102229 |
| LIPSIA (2012) \| .7908532 .61040342 1.0246483 |
| ELISA3 (2013) \| .71007735 .52863741 .9537915 |
| Liu et al. (2015) \| .81884605 .63114542 1.0623683 |
| RIDDLE (2015) \| .80982172 .62417167 1.0506905 |
| SISCA (2015) \| .77661043 .59927207 1.0064274 |
| -------------------+---------------------------------------------------------- |
| Combined \| .78640021 .60792267 1.0172763 |
| ------------------------------------------------------------------------------ |

**Figure.5 Sensitivity analysis plot of repeated revascularization between early invasive strategy and delayed invasive strategy.**


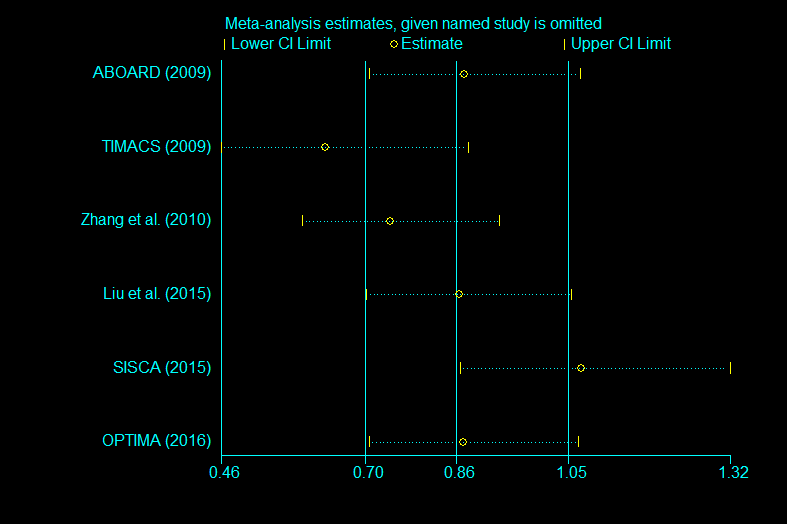


| ------------------------------------------------------------------------------ |
| --- |
| Study omitted \| Estimate [95% Conf. Interval] |
| -------------------+---------------------------------------------------------- |
| ABOARD (2009) \| .87169498 .71114081 1.0684974 |
| TIMACS (2009) \| .6354447 .45903954 .87964094 |
| Zhang et al. (2010)\| .74620497 .59749424 .9319284 |
| Liu et al. (2015) \| .86297673 .70634586 1.0543401 |
| SISCA (2015) \| 1.0699259 .86480945 1.3236921 |
| OPTIMA (2016) \| .8702234 .71057284 1.065744 |
| -------------------+---------------------------------------------------------- |
| Combined \| .85878788 .70342925 1.0484588 |
| ------------------------------------------------------------------------------ |

**Figure.6 Sensitivity analysis plot of mortality rate between immediate invasive strategy and delayed invasive strategy.**


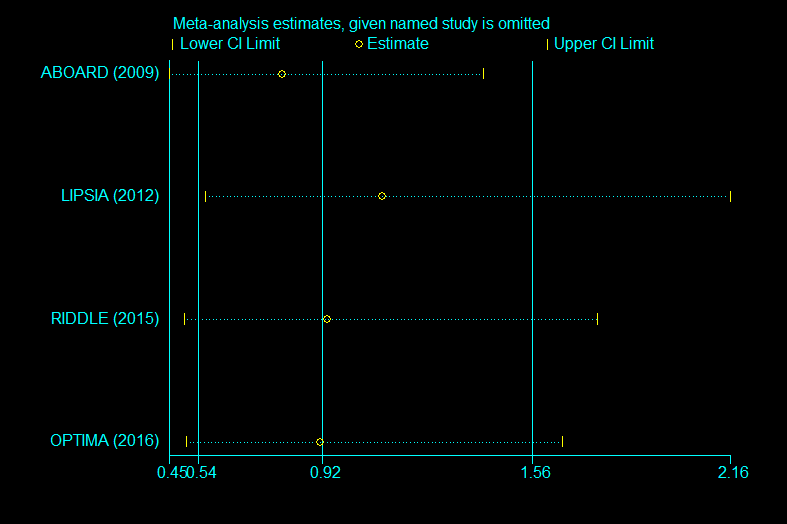


| ------------------------------------------------------------------------------ |
| --- |
| Study omitted \| Estimate [95% Conf. Interval] |
| -------------------+---------------------------------------------------------- |
| ABOARD (2009) \| .79779226 .45150962 1.4096543 |
| LIPSIA (2012) \| 1.1019492 .56207871 2.1603594 |
| RIDDLE (2015) \| .93511575 .49795425 1.7560679 |
| OPTIMA (2016) \| .91221285 .50440407 1.6497337 |
| -------------------+---------------------------------------------------------- |
| Combined \| .9177748 .54035943 1.5587969 |
| ------------------------------------------------------------------------------ |

**Figure.7 Sensitivity analysis plot of myocardial infarction between immediate invasive strategy and delayed invasive strategy.**


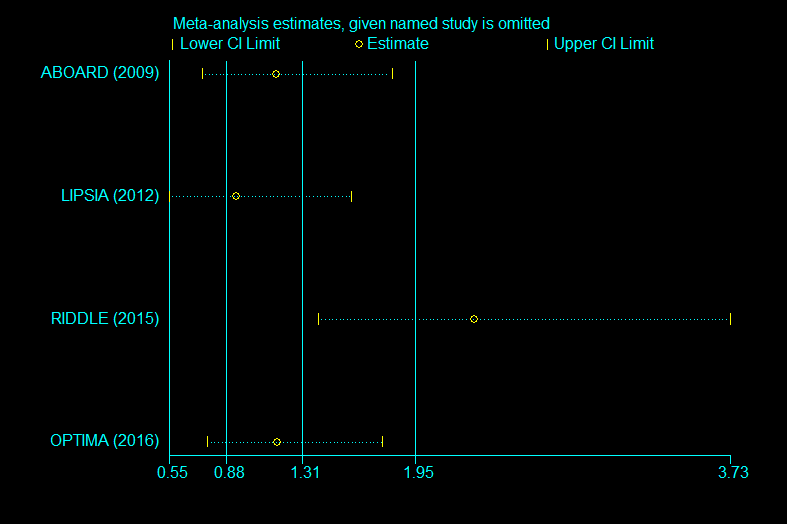


| ------------------------------------------------------------------------------ |
| --- |
| Study omitted \| Estimate [95% Conf. Interval] |
| -------------------+---------------------------------------------------------- |
| ABOARD (2009) \| 1.1608313 .74153483 1.8172165 |
| LIPSIA (2012) \| .93730509 .55437046 1.5847541 |
| RIDDLE (2015) \| 2.2840426 1.3986241 3.7299881 |
| OPTIMA (2016) \| 1.1663102 .77270949 1.7604021 |
| -------------------+---------------------------------------------------------- |
| Combined \| 1.3100028 .88119733 1.9474721 |
| ------------------------------------------------------------------------------ |

**Figure.8 Sensitivity analysis plot of refractory ischemia between immediate invasive strategy and delayed invasive strategy.**


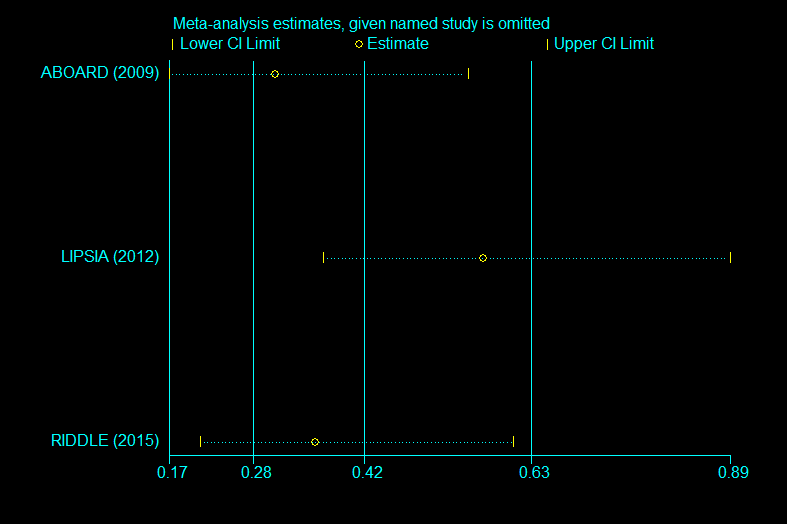


| ------------------------------------------------------------------------------ |
| --- |
| Study omitted \| Estimate [95% Conf. Interval] |
| -------------------+---------------------------------------------------------- |
| ABOARD (2009) \| .30595762 .16927694 .5529995 |
| LIPSIA (2012) \| .57160777 .3677049 .88858062 |
| RIDDLE (2015) \| .35744914 .20941542 .61012644 |
| -------------------+---------------------------------------------------------- |
| Combined \| .41916783 .27711845 .63403095 |
| ------------------------------------------------------------------------------ |

**Figure.9 Sensitivity analysis plot of major bleeding between immediate invasive strategy and delayed invasive strategy.**


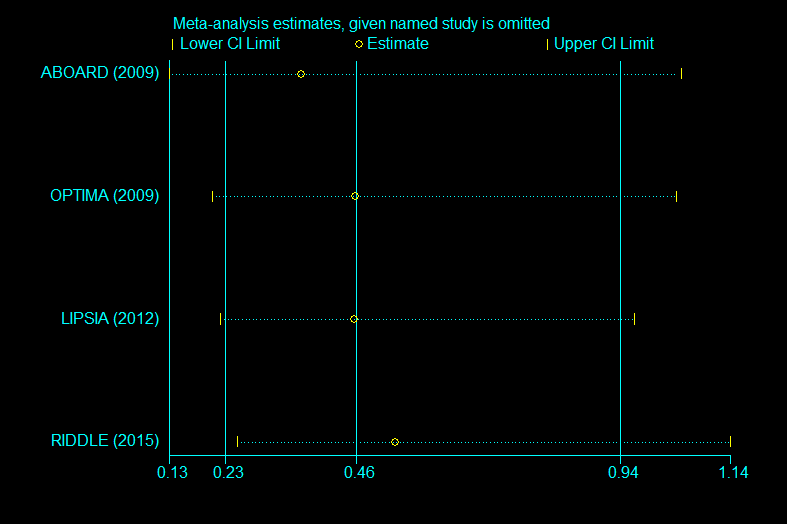


| ------------------------------------------------------------------------------ |
| --- |
| Study omitted \| Estimate [95% Conf. Interval] |
| -------------------+---------------------------------------------------------- |
| ABOARD (2009) \| .36644575 .12812053 1.0480951 |
| OPTIMA (2009) \| .46222878 .20587599 1.037787 |
| LIPSIA (2012) \| .461133 .22092584 .96251142 |
| RIDDLE (2015) \| .53419703 .25129181 1.1355981 |
| -------------------+---------------------------------------------------------- |
| Combined \| .46412085 .22961656 .93812119 |
| ------------------------------------------------------------------------------ |

**Figure.10 Sensitivity analysis plot of repeated revascularization between immediate invasive strategy and delayed invasive strategy.**


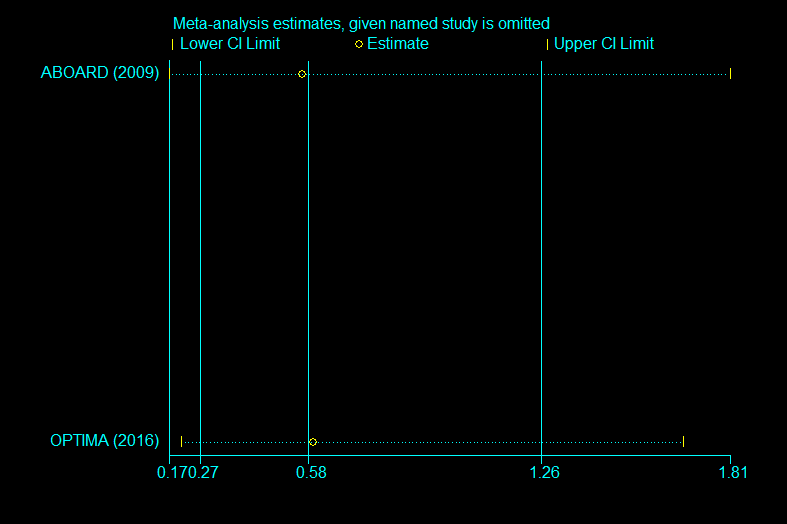


| Study omitted \| Estimate [95% Conf. Interval] |
| --- |
| -------------------+---------------------------------------------------------- |
| ABOARD (2009) \| .56066179 .17408222 1.8057077 |
| OPTIMA (2016) \| .59289938 .21072577 1.6681857 |
| -------------------+---------------------------------------------------------- |
| Combined \| .57859216 .26657454 1.2558172 |
| ------------------------------------------------------------------------------ |

**Figure.11 Sensitivity analysis plot of major bleeding between immediate (<6h) invasive strategy and delayed invasive strategy.**


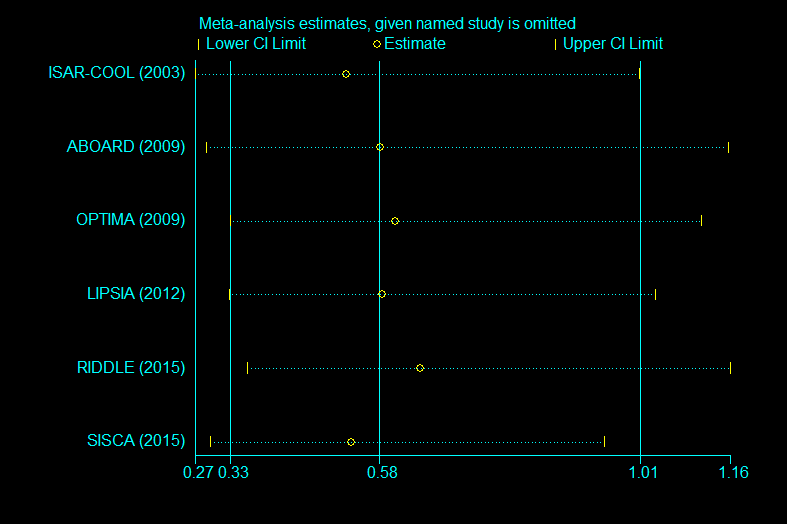


| ------------------------------------------------------------------------------ |
| --- |
| Study omitted \| Estimate [95% Conf. Interval] |
| -------------------+---------------------------------------------------------- |
| ISAR-COOL (2003) \| .52194667 .26906767 1.0124899 |
| ABOARD (2009) \| .578978 .28870824 1.1610875 |
| OPTIMA (2009) \| .60459834 .32755104 1.1159763 |
| LIPSIA (2012) \| .58196998 .32622284 1.0382137 |
| RIDDLE (2015) \| .64506733 .3574805 1.1640128 |
| SISCA (2015) \| .53086329 .29547206 .95378178 |
| -------------------+---------------------------------------------------------- |
| Combined \| .57687313 .32862172 1.0126616 |
| ------------------------------------------------------------------------------ |

TSA was done to evaluate the sample size and correct errors in the comparision assessing refractory ischemia between early (<24h) and delayed invasive therapy. The conventional boundary and TSA curve has been crossed, indicating that the cumulative evidence is conclusive. [Fig.1]


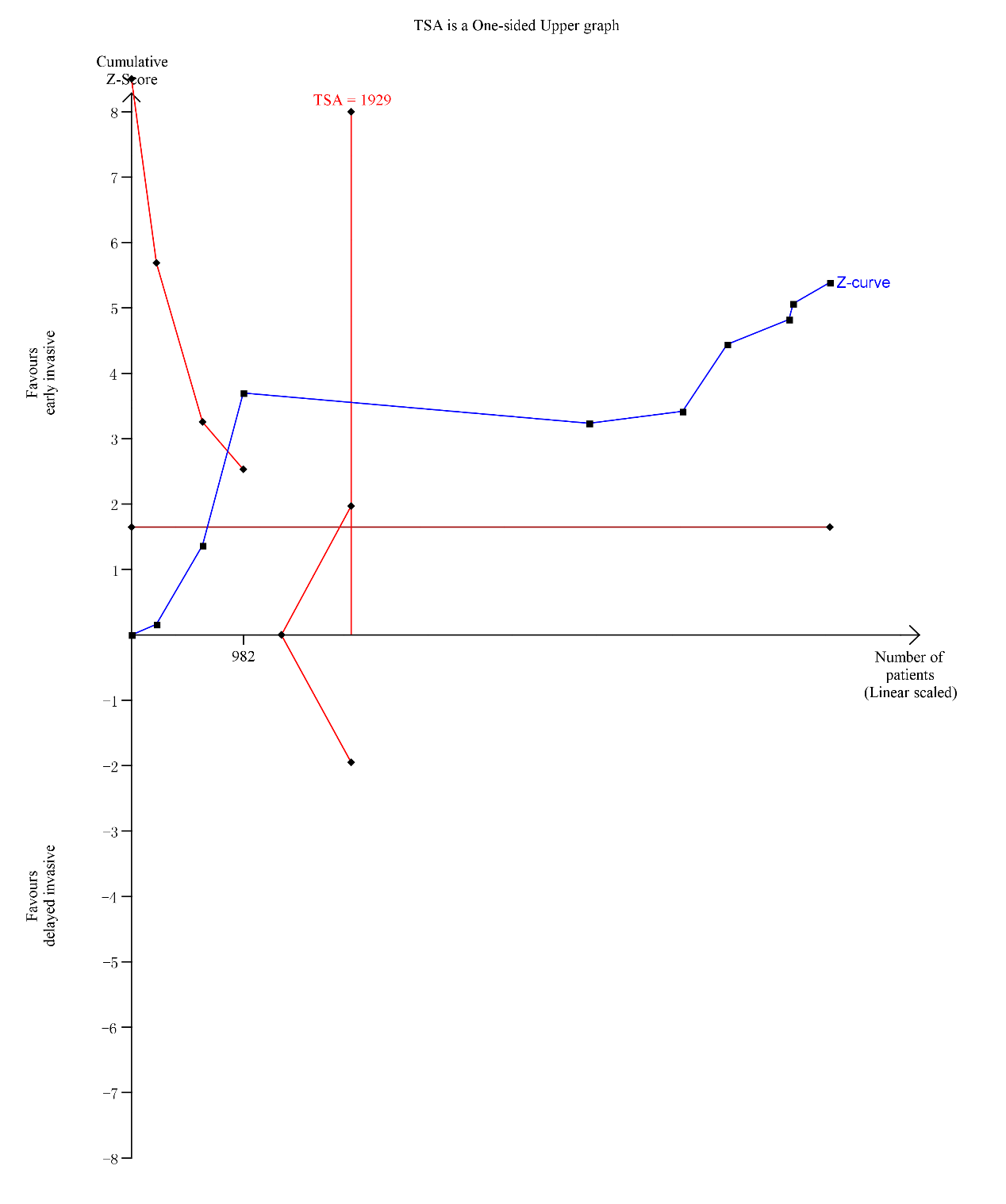


**Figure 12.** TSA plot for the comparision assessing refractory ischemia between early (<24h) and delayed invasive therapy

TSA was done to evaluate the sample size and correct errors in the comparision assessing major bleeding between immediate (<2h) and delayed invasive therapy. The conventional boundary and TSA curve has been crossed, indicating that the cumulative evidence is conclusive. [Fig.2]


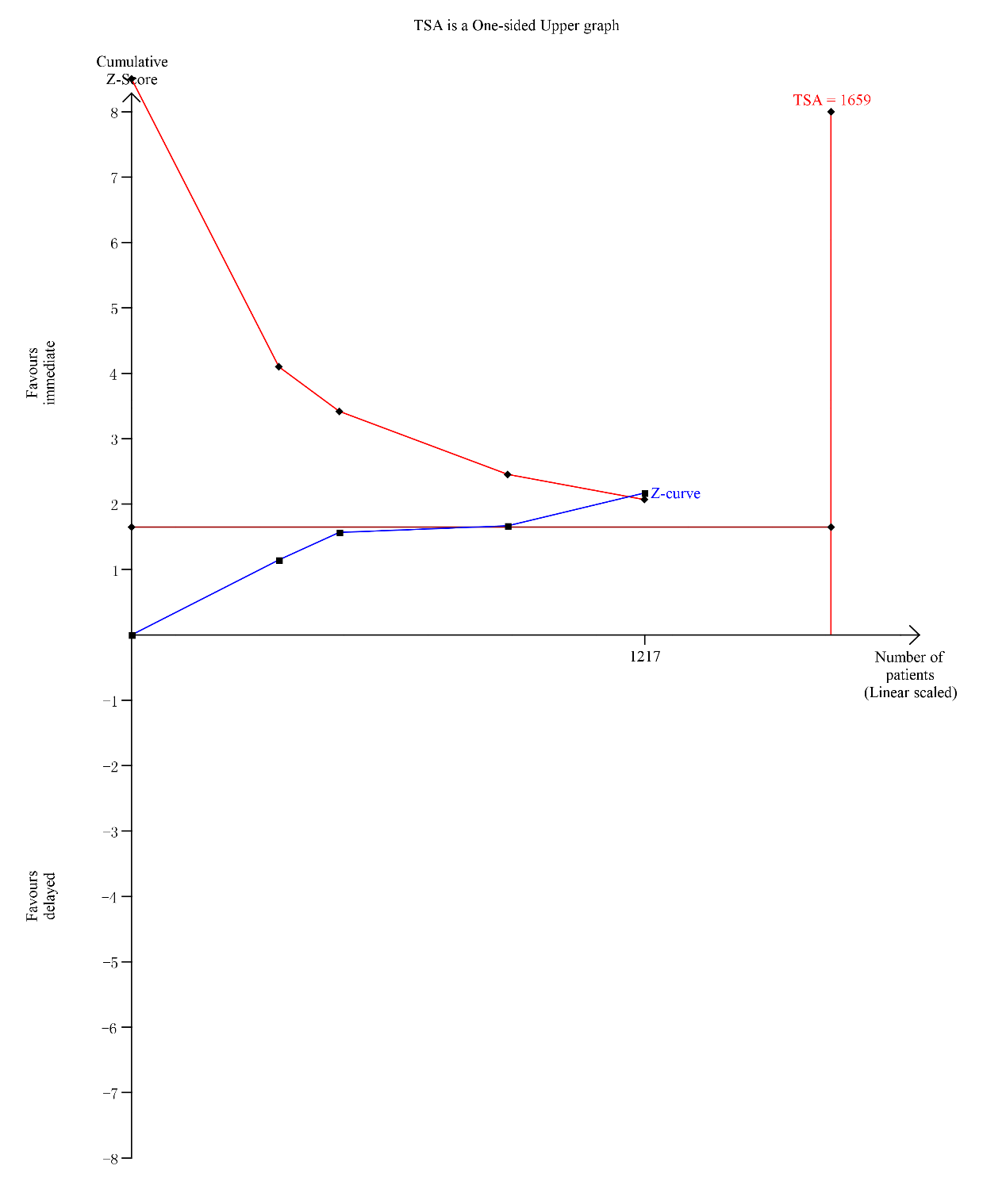


**Figure 13.** TSA plot for the comparision assessing major bleeding between immediate (<2h) and delayed invasive therapy
